# Review of Statistical Methods for Analysing Healthcare Resources and Costs

**DOI:** 10.1002/hec.1653

**Published:** 2010-08-26

**Authors:** Borislava Mihaylova, Andrew Briggs, Anthony O'Hagan, Simon G Thompson

**Affiliations:** a Health Economics Research Centre, University of OxfordOxford, UK; b Public Health and Health Policy, University of GlasgowGlasgow, UK; c Department of Probability and Statistics, University of SheffieldSheffield, UK; d MRC Biostatistics UnitCambridge, UK

**Keywords:** healthcare costs, healthcare resource use, randomised trials, statistical methods

## Abstract

We review statistical methods for analysing healthcare resource use and costs, their ability to address skewness, excess zeros, multimodality and heavy right tails, and their ease for general use. We aim to provide guidance on analysing resource use and costs focusing on randomised trials, although methods often have wider applicability. Twelve broad categories of methods were identified: (I) methods based on the normal distribution, (II) methods following transformation of data, (III) single-distribution generalized linear models (GLMs), (IV) parametric models based on skewed distributions outside the GLM family, (V) models based on mixtures of parametric distributions, (VI) two (or multi)-part and Tobit models, (VII) survival methods, (VIII) non-parametric methods, (IX) methods based on truncation or trimming of data, (X) data components models, (XI) methods based on averaging across models, and (XII) Markov chain methods. Based on this review, our recommendations are that, first, simple methods are preferred in large samples where the near-normality of sample means is assured. Second, in somewhat smaller samples, relatively simple methods, able to deal with one or two of above data characteristics, may be preferable but checking sensitivity to assumptions is necessary. Finally, some more complex methods hold promise, but are relatively untried; their implementation requires substantial expertise and they are not currently recommended for wider applied work. Copyright © 2010 John Wiley & Sons, Ltd.

## 1. INTRODUCTION

It is well recognized that statistical analysis of healthcare resource use and cost data poses a number of difficulties. These non-negative data often exhibit substantial positive skewness, can have heavy tails and are often multimodal (e.g. with a mass at zero for non-users). The traditional approach for handling such non-normal data in medical statistics has been to use non-parametric methods, such as rank order statistics. Nevertheless, it is widely accepted in health economics that it is the estimated population mean cost that is the statistic of interest to policy makers (Arrow, 1970). Two broad areas have emerged for the statistical modelling of cost data.

In the ‘randomized evaluation’ field, healthcare resource use and cost data are collected, often alongside randomised controlled trials of limited sample size, in order to study the impact of interventions or policies on average costs in terms of mean cost difference, while minimising bias. These studies are used to evaluate the effectiveness, cost-effectiveness and other effects of interventions and guide treatment and policy decisions. In general, more complex approaches for the analysis of mean costs in such studies that take into consideration the specific features of the data might lead to gains in precision and to more informative estimates, but could run a risk of misfitting or overfitting the data. The ‘health econometrics’ field is characterised by the use of large quantities of mostly observational data to model individual healthcare expenditures, with a view to understanding how the characteristics of the individual, including their health status or recent medical experience, influence overall costs. A theoretical behaviour model might be used within the framework to shed light on processes in action. Observational data are vulnerable to biases in estimating effects due to non-random selection and confounding that are avoided in randomised experimental data.

The literature on evaluating costs for the purpose of cost-effectiveness analysis and that on health econometrics have developed largely independently. This paper provides a novel review of the analytical approaches to evaluating mean resource use and costs and mean cost differences employed in both areas, with a particular focus on their applicability to mean cost differences estimated in randomised trials. Although the fundamental interest relates to the raw cost scale, analysis can be performed on a different scale for the purposes of estimation provided a mechanism exists for returning to the original cost scale. The objective is to examine the state-of-the-art of statistical analysis of healthcare resource use and cost data, by identifying the methods employed, their ability to address the challenges of the data and their ease for general use. Based on this review, we propose a framework to guide researchers when analysing resource use and costs in clinical trials. We exclude from this review methods for estimation of behavioural economic models or adjusting for selection bias and measurement errors. An overview of health economics estimation strategies using individual level data and microeconometric techniques is provided elsewhere (Jones, 2000).

Previous work has noted the lack of a dominant approach that provides both unbiased and efficient estimates (Manning and Mullahy, 2001) and the need to perform checks and validations to find a suitable model (Buntin and Zaslavsky, 2004). Recently, Basu and Manning (2009) concluded, ‘No current method is optimal or dominant for all cost applications. Many of the diagnostics used in choosing among alternatives have limitations that need more careful study. Several avenues in modeling cost data remain unexplored.’ In our more comprehensive and systematic review, we come to broadly similar conclusions, but we also go further and offer some general advice to researchers on the choice of methods when estimating mean costs.

## 2. REVIEW METHODS

### 2.1. Identification of papers for inclusion in the review

The review aims to identify the analytical methods currently employed or suggested for evaluating healthcare resource use and costs that are likely to be applicable to randomised trial data. A number of exclusion criteria were employed in order to limit the scope of what might potentially include a very large literature.

1. As the focus of the review was methodological, we did not aim to incorporate all applications of these methods to different data sets but important modifications were reviewed.
2. Analytical methods developed to account for selection bias, unobserved heterogeneity or measurement error were considered beyond the scope of this review.
3. Methods that do not allow for (or do not focus on) the estimation of the mean costs or mean cost differences (between interventions) on the untransformed scale were also excluded.
4. Methods aimed to address particular aspects of evaluating costs alongside multinational or multi-centre trials were not reviewed.

Analytical methods that account for administrative censoring and missing data, which are usually present in randomised trials, are not explicitly reviewed but are briefly summarised in a web appendix (http://www.herc.ox.ac.uk/downloads/support_pub).

**Figure 1:**
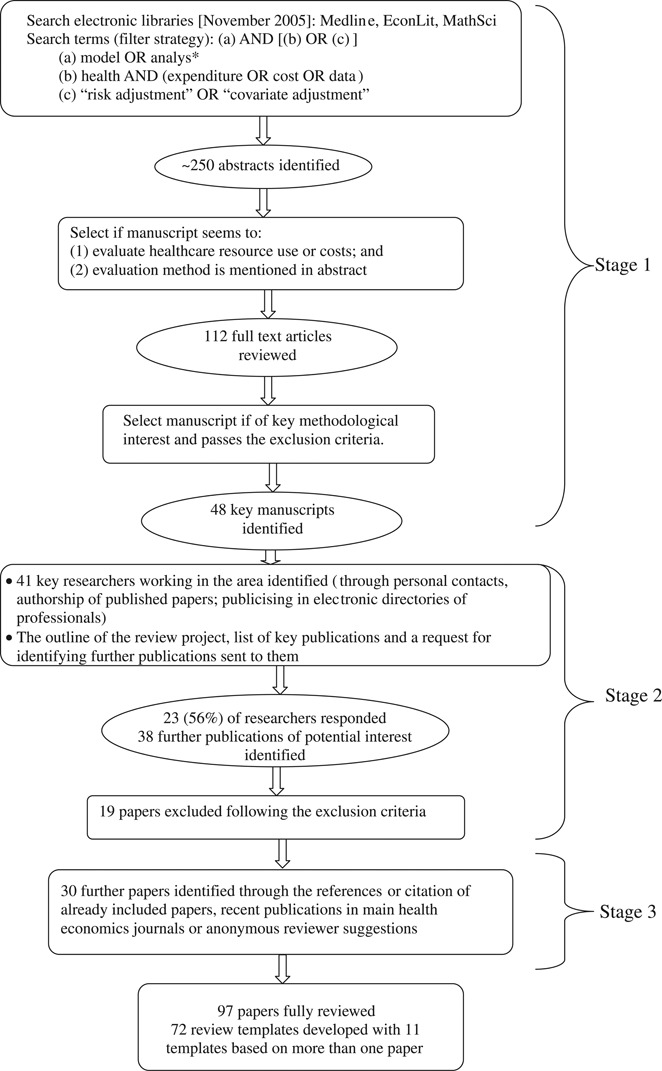
Flow chart of selection of papers into the review

The three stage process employed to identify the key publications to include in this review is outlined in Figure 1. First, a broad search of Medline, EconLit and MathSci bibliographic databases generated 250 publications of possible methodological interest. Applying the criteria above reduced these to 48 key publications. In the second stage, a brief outline of the review objectives together with the list of these key publications were sent to 41 individuals working in the area (identified through known contacts and posting to relevant email discussion lists such as healthecon-all@jiscmail.ac.uk) with a request to suggest further published research for inclusion in the review. In all, 23 researchers responded and suggested a further 38 publications of potential interest of which 19 met the inclusion criteria (9 papers by one author were replaced with a subsequent review paper by the same author). In the final stage, a review of the citations from the studies included in the review and searching recent issues of key journals yielded a further 30 studies for inclusion. In total 97 manuscripts were therefore included in the review. No explicit quality criteria for the studies reviewed was employed as our aim was to identify all relevant suggested methods.

### 2.2. Review process

The identified papers were reviewed by two of the authors (B. M. initially reviewed all papers and each of the remaining authors reviewed a proportion of the papers). A template summarizing the relevant details for each paper was completed. In total 72 review templates were developed, of which 11 covered more than one paper. Manuscripts by the same author(s) presenting methods from the same category were summarised in a single template. Each template aimed to present a structured factual review of the paper(s) focusing on the method(s) employed, data used and overall methodological findings and conclusions. The summarised details included (a) the parameters of interest and the representation of the estimation error; (b) statistical model and estimation method(s), (c) ease of implementation, (d) characteristics of the applied or simulated data used (including sample size) and (e) the authors' conclusions with respect to the analytical method used. In addition, the review authors tried to judge the ability of the methods to (1) address skewness, multimodality and kurtosis in data; (2) incorporate adjustment for covariates and extend to cost-effectiveness analysis and (3) be applicable to data sets of small to moderate sample sizes usually available in randomised studies, where these issues were not explicitly considered by the authors themselves.

## 3. REVIEW RESULTS

### 3.1. Categorising the analytical approaches

Based on the reviewed papers, 12 categories of analytical approaches currently employed to evaluate mean healthcare resource use and costs were identified. These categories are outlined below together with a brief description. The review templates are available in the web appendix (http://www.herc.ox.ac.uk/downloads/support_pub).

#### 3.1.1. Normal distribution-based methods

Methods based on the normal distribution are widely employed in the estimation of mean healthcare resource use and costs. They include inference based on the sample mean (such as the *t*-test) and linear regression approaches (such as ordinary least squares, OLS). These methods present results on the scale of interest and provide unbiased estimates for randomised data. However, as illustrated in comparative studies, they are sensitive to extreme values and likely to be inefficient in small to medium sample sizes if the underlying distribution is not normal (Austin *et al*., 2003; Basu, 2005; Briggs *et al*., 2005; Briggs and Gray, 1998; Chen and Zhou, 2006; Deb and Burgess, 2003; Dinh and Zhou, 2006; Duan *et al*., 1983; Dudley *et al*., 1993; Gilleskie and Mroz, 2004; O'Hagan and Stevens, 2003; Thompson and Barber, 2000; Zhou, 2002; Zhou and Dinh, 2005). Generalised least squares estimators or Huber/White estimate of the variance–covariance matrix for OLS regression are often used to achieve consistent estimates in such situations (Manning, 1998).

#### 3.1.2. Methods following transformation of data

For these methods the individual healthcare resource use or cost data is transformed in order to overcome problems of skewness and comparison of means on the transformed scale (Briggs and Gray, 1998) or OLS type approaches (as above) to model the transformed dependent variable are employed (Ai and Norton, 2000; Duan *et al*., 1983; Manning and Mullahy, 2001; Veazie *et al*., 2003). Although more precise and robust, these comparisons of means on the transformed scale do not directly inform the comparison of means on the original scale and back transformation of the results to the original scale is required (Duan, 1983).

Approaches to find an appropriate transformation have centred predominantly on the Box–Cox transformations (to achieve symmetry in error), with log transformation often prevailing when modelling costs. The power of transformation is commonly selected based on the data, but treated as known when estimating the model parameters, which is likely to affect the confidence interval for the mean estimates. Taylor (1986) has shown that using the estimated value for the power (with its uncertainty) is feasible in most cases, and will provide more accurate confidence intervals; the price in precision is likely to be small compared to assuming the power known. When the power is estimated close to zero with large variance, however, more precision is lost. A Bayesian approach incorporating estimation for the power has also been suggested (Hollenbeak, 2005).

The approaches for back transformation to the original scale are dictated by the nature of the error term on the transformed scale. Under the assumption of normality of the error term, the mean on the untransformed scale can be directly estimated from the estimates of model parameters and variance. The distribution of the error term is however usually unknown, and reliance on the assumption of normality or homoskedasticity can lead to inconsistent estimates. Thus, the approaches for back transformation are dominated by Duan's non-parametric smearing when the error term is homoskedastic (Duan, 1983) and its variants when the error term is heteroskedastic across the compared groups (Ai and Norton, 2000; Duan *et al*., 1983; Manning, 1998). Alternative approaches of direct numerical integration over fully parameterized error distributions are proposed by Abrevaya (2002) and Ai and Norton (2000), while Ai and Norton (2002) propose a more general semi-parametric approach.

The methods employing an initial data transformation are shown to provide potentially more efficient estimates in heavy-tailed data (Manning and Mullahy, 2001; O'Hagan and Stevens, 2003), but can perform badly if an inappropriate transformation is used (Briggs *et al*., 2005). Certain transformations (e.g. logarithmic) are also not appropriate for zero data. Adding a constant to the zero responses is generally not recommended: first the constant chosen is somewhat arbitrary, second it does not acknowledge that the observed zeros might be influenced differently by covariates than the positive responses and third it has been shown to perform poorly with some evidence for overfitting (e.g. mishandling of both non-users and significant users of health services) (Duan *et al*., 1983).

#### 3.1.3. Single distribution generalized linear models

In generalized linear models (GLMs) a mean function (between the linear predictor and the mean) and a variance function (between the mean and variance on the original scale) are specified and the parameters are estimated given these structural assumptions (see general theory in Blough *et al*. (1999)). This approach addresses linearity in response on the specified scale and accommodates skewness through variance weighting. Although misspecification of the variance function could lead to inefficiencies, the mean function estimates are usually robust (Manning and Mullahy, 2001). As the estimation is directly on the scale of raw data, unlike the transformation-based approaches, there is no need for back transformation. These models are used both for modelling costs (e.g. Gamma specification) as well as resource use (e.g. Poisson and negative binomial specifications). Extensions of the standard GLM approach are provided based on extending the family of distributions (Manning *et al*., 2005), more complex specification of the mean–variance relationships (Basu, 2005; Basu *et al*., 2006; Blough *et al*., 1999) and GLM estimators that are more robust to outliers (and potentially more suitable for very heavy-tailed data) (Cantoni and Ronchetti, 2001; Cantoni and Ronchetti, 2006). GLMs are also used in two-part models to model the positive resource use and cost data (see Section 3.1.6 below). The most widely used GLM model with log link has been shown to suffer substantial efficiency losses when the log-scale error variance is large or the error distribution on the log scale is symmetric but heavy-tailed, but is more efficient for alternative shapes of the distribution (Manning and Mullahy, 2001). Heteroskedasticity in the error variance on the log-scale can also be problematic in a GLM model if not properly modelled. For a guide to analysts on how to choose among exponential conditional mean models see Manning and Mullahy (2001), Buntin and Zaslavsky (2004) and Manning *et al*. (2005). The Generalized Gamma model (Manning *et al*., 2005) also extends the shapes of distributions (e.g. Weibull and OLS for the lognormal models are included) and provides further tests of appropriateness of GLM or OLS following log transformation. Furthermore, although GLM model specifications could be informed by the data (Blough *et al*., 1999), the estimation for the link and variance and the incorporation of the uncertainty from this estimation (including underlying heteroscedasticity) in the final parameter estimates is rarely done; for an exception see the Extended Estimating Equations approach in Basu and Rathouz (2005).

#### 3.1.4. Parametric models based on skewed distributions outside the GLM family of distributions

Methods based on distributions outside the GLM family have been used to improve flexibility of previous parametric models while modelling healthcare costs or resource use. Two and three-parameter lognormal and log-logistic distributions are used by Nixon and Thompson (Nixon and Thompson, 2004; Thompson and Nixon, 2005) to model costs, and lognormal and Weibull models are used by Marazzi *et al*. (1998) to model length of stay in hospital. General methods for count data have been extensively described elsewhere (Cameron and Trivedi, 1998; Winkelmann, 2008). Extended approaches based on the Poisson distribution by Cameron and colleagues (Cameron and Johansson, 1997; Cameron and Trivedi, 1986) (negative binomial and variance linear in the mean, Poisson polynomial model) and Grootendorst (1995) (zero-inflated Poisson and negative binomial) are used to model resource use. The zero-inflated Poisson and negative binomial specifications have been shown to suffer convergence problems if the same covariates are used for both parts (Grootendorst, 1995). These further parametric alternatives aim to achieve better fit to data (e.g. in terms of deviance) but studies failed to confirm that better fit translates into more reliable inferences potentially due to models over-fitting extreme observations (Nixon and Thompson, 2004; Thompson and Nixon, 2005).

#### 3.1.5. Models based on mixture of parametric distributions

Models based on mixtures of parametric models are introduced as a flexible way to accommodate excess zeros, overdispersion and heavy tails that might lead to more robust estimates. A further motivation for mixture models is the concern that different parts of the response distribution could be differently affected by covariates (i.e. rare users, low cost users, high cost users). Mixtures of Poisson distributions (Mullahy, 1997) and negative binomial distributions (Deb and Holmes, 2000; Deb and Trivedi, 1997; Jimenez Martin *et al*., 2002) have been suggested for resource use data, and mixtures of Gamma distributions for cost data (Deb and Burgess, 2003). The papers presenting applications of mixture models on cost data have focused on modelling positive cost data, while modelling excess zeros is likely to present a problem. A mixture of distributions from different families has been shown to improve on a mixture of distributions from the same family (Atienza *et al*., 2008). Mixture models often perform better than model alternatives based on single distributions for total resource use or costs. Computational difficulties can arise as often the log likelihood function has multiple maxima and choosing a suitable optimisation procedure is needed. Furthermore in the case of unknown separation (latent mixture models) and more than two components the identification of all components might be difficult because of the increasing overlap of distributions (Cameron and Trivedi, 2009). More robust estimation procedures via alternatives to maximum likelihood have been recently suggested (Lu *et al*., 2003). Mixture models with known separation (e.g. two-part models in the subsequent category) do not exhibit such estimation issues.

#### 3.1.6. Two *(*or multi*)*-part models *(*also hurdle models for count data*)* and Generalised Tobit models

The two-part model is a special case of a mixture model in which only two components are allowed and one of these is degenerate (a probability distribution whose support consists of only one value); these components (unlike mixture models) are separately and independently estimated. These models have been widely employed in situations where, due to large numbers of non-users of health services, there are excess zeros in the resource use or cost data and the assumption of normality of the error term is not satisfied. Usually, a logit or probit model for the first part estimates the probability of incurring any resource use or costs, while the mean resource use or costs, conditional on having incurred any, are evaluated in the second part. Log-linear (Duan *et al*., 1983; Leung and Yu, 1996), GLM or OLS models have been employed in the second part to evaluate mean costs, and truncated-at-zero Poisson, negative binomial (Grootendorst, 1995; Pohlmeier and Ulrich, 1995), or truncated Poisson-lognormal models (Winkelmann, 2004) to evaluate resource use (for a wider range of hurdle models please refer to Hilbe (2007)).

Extensions to the standard two-part model have been identified in our review. A Weibull survival model was suggested for the hazard of initiating mental health service use as duration on treatment (time from initiating treatment to end of year) was indicative of cost (Keeler *et al*., 1988). Santos-Silva and Windmeijer suggest more appropriate modelling of spells of care and number of visits per spell through a Poisson model for the first part (number of spells) and a logarithmic model for the second part (number of visits per spell) (Santos-Silva and Windmeijer, 2001). A semi-parametric two-part model for count regression (including Poisson or negative binomial hurdle models as a special case) is suggested by Gurmu (1997), and can be extended through a semi-parametric single-index two-part regression model (Zhou and Liang, 2006). A hurdle model with a generalised logistic model in the first part is proposed by Gurmu (1998) and a modified second part in the two-part model is suggested by Mullahy (1998). A Bayesian implementation of a two-part model in WinBUGS is illustrated by Cooper *et al*. (2003). Another extension uses a Poisson-lognormal model to improve flexibility when modelling positive outcomes (Winkelmann, 2004). A four-part specification was proposed by Duan *et al*. (1983) for different resources use categories, and motivates the discussion on how many parts are needed. The authors show that modelling different components of resource use improves the consistency of the model without issues of data overfitting, but does not improve efficiency compared to a two-part model, presumably due to insufficient sample size. The specification of this four-part models also might be considered an example of data components models (see Section 3.1.10 below).

Two-part models are shown to perform better than single-equation models in terms of split sample mean-squared forecast error as they accommodate heterogeneity between users and non-users as well as heterogeneity across users based on level of use (Duan *et al*., 1983), but mixture models are suggested to be more appropriate if distinct population groups contribute to different patterns of use (Deb and Holmes, 2000). Also, when transformation of data is employed, the need for back transformation to the original scale emerges and is exacerbated in the case of two-part models due to added conditionality. In this regard, Mullahy (1998) proposes two estimators using exponential conditional mean specifications. An extension of Duan's smearing estimate which combines the two parts of the two-part model, with explicit accounting for heteroscedasticity and zero observations, to produce mean estimates on the original scale is developed by Welsh and Zhou (2006).

Although we explicitly exclude selection models from the current review (as zeros are genuine observations rather than unobserved), we will discuss the generalized Tobit model that allows for correlation between the propensity to use healthcare services and the level of use. Also, unlike the sample selection model, it acknowledges as genuine the zero data points (van de Ven and Van Praag, 1981) and, unlike the Tobit model, allows for different covariates in the two parts, bivariate normal error term and possibly a transformation for the positive outcome part. A debate on the superiority of two-part or selectivity models has developed following the use of two-part models in the RAND Health Insurance Experiment (HIE) (Duan *et al*., 1983). Hay and Olsen (1984) argue that if the two parts are not independent the conditional error distribution in the first part will be a function of covariates and should be made explicit in the estimation. The benefits of this proposition have not been observed in the case of the RAND HIE and are unlikely to hold generally in randomised studies when the interest is in the estimated mean (Duan *et al*., 1984; Maddala, 1985; Manning *et al*., 1987). It has also been shown that co-linearity problems and violation of the bivariate normality assumption for the error term, likely in health data sets, lead to poor performance of selectivity models and the two-part model is likely more efficient (Leung and Yu, 1996; Manning *et al*., 1987). An extended two-part model, which explicitly models and estimates the correlation between the logistic and lognormal part for repeated measures data, is suggested by Tooze (Tooze *et al*., 2002) and is shown to outperform the model with uncorrelated random effects.

#### 3.1.7. Survival *(*or duration*)* methods

General methods to adjust for censoring are not explicitly reviewed here, but are briefly summarised with key references in the web appendix. Survival data models, considering cost as the ‘time’ variable, can relax the parametric assumptions of the exponential conditional mean models, and might be advantageous particularly for data with skewness, heavy tails and multimodality and are thus reviewed here. Survival data-based approaches. such as the semi-parametric Cox proportional hazards model (Austin *et al*., 2003; Basu *et al*., 2004; Dudley *et al*., 1993; Lipscomb *et al*., 1998) and the parametric Weibull proportional hazards model (Dudley *et al*., 1993) have been shown to perform well when the underlying proportional hazards assumption is met (Austin *et al*., 2003; Dudley *et al*., 1993) but produce biased estimates otherwise (Basu *et al*., 2004). Basu and Manning (2006) proposes a test for the proportional hazards assumption within the class of exponential conditional mean models that performs similarly to a traditional test based on the Cox proportional hazards regression.

Another survival type approach suggested in the literature is the Aalen regression model with additive hazard function (Pagano *et al*., 2008). This method does not require the proportionality in hazards (an assumption imposed in the Cox and Weibull proportional hazards models), conveniently preserves the additivity of the covariates' effects on the outcome measure and can perform well in large data sets with censoring.

#### 3.1.8. Non-parametric methods

Two common approaches to directly evaluate uncertainty in costs, effects and/or cost-effectiveness in randomised trials are the Central Limit Theorem and the bootstrap approach (Barber and Thompson, 2000; O'Hagan and Stevens, 2003; Thompson and Barber, 2000). While the first approach relies on normality of sample mean costs and/or effects with large number of participants, independently of the population distribution of costs and/or effects, the latter uses empirical estimation of the sampling distribution of mean costs and/or effects by resampling from the data set with replacement and re-estimating the sample means for each replicate. Both approaches are asymptotically valid as sample sizes increase, but have uncertain properties in smaller non-normally distributed data samples. Different bootstrap procedures are compared by Barber and Thompson (2000) who suggest that some bootstrap approaches (bootstrap-t and bias corrected bootstrap) might be more reliable than others. O'Hagan and Stevens (2003) criticize the bootstrap and the Central Limit Theorem approaches as inefficient for use with skewed healthcare cost data and caution against their use with small data sets. A recent study which compared the performance of the two approaches concluded that they both provide accurate mean estimates even in relatively small samples from skewed distributions, with the Central Limit Theorem-based methods providing at least as accurate estimates of standard errors as the bootstrap (Nixon *et al*., 2009). Approaches based on Edgeworth expansion of the *t*-test or a modification of *t*-test based on a generalized pivotal statistic add flexibility to account for skewness and have been shown to provide more efficient estimates but these modifications are subject to a degree of subjectivity in the choice of transformation and do not generally allow for adjustment for covariates (Chen and Zhou, 2006; Dinh and Zhou, 2006; Zhou and Dinh, 2005).

Quantile-based smoothing methods can improve efficiency by borrowing strength from similarities in data sets, but assuming such similarities could also lead to biases (Dominici and Zeger, 2005). Regression of the quantiles of costs (Wang and Zhou, 2010) has been shown to have competitive and more robust performance compared to the smooth quantile ratio estimator (Dominici *et al*., 2005), maximum likelihood estimator, generalised linear models based on quasilikelihood (Blough and Ramsey, 2000), and the internal weighted estimator (Welsh and Zhou, 2006) in various heteroscedastic models. A non-parametric method based on a discrete approximation of the density function of the outcome using a sequence of conditional probability density functions is shown to provide robust conditional mean estimates, at least in large data sets, when mass at zero or multimodality is present in data or when covariate effects differ over ranges of the outcome (Gilleskie and Mroz, 2004). A semi-parametric mixture that models the bulk of the data (non-extreme values) with piecewise constant densities and the tails (extreme values) with a generalised Pareto distribution while taking into account the tail model uncertainty provides further flexibility to model well data with very different shapes (including multimodal data), but might not be efficient if only sparse data are available in the tail of the distribution (Conigliani and Tancredi, 2005).

#### 3.1.9. Methods based on truncation or trimming of data

A series of papers by Marazzi (Marazzi, 2002; Marazzi and Barbati, 2003; Marazzi and Ruffieux, 1999; Marazzi and Yohai, 2004) illustrates the use of truncation to provide more robust estimates of the mean. Data are modelled using parametric distributions, which are subsequently truncated from both ends (to discard contaminants) in such a way as to preserve the mean of an underlying uncontaminated distribution. A degree of robustness is claimed under moderate contamination. The approach is based on the important assumption that the data are contaminated (in particular with high values) that is not appropriate in the case of healthcare resource use and costs where zero or high observations are true values. Therefore, these approaches can lead to substantial bias in estimation of the mean.

#### 3.1.10. Data components models

An emerging area of research is analysis in which components of resource use or costs are modelled separately under a common analytical framework. The applications published to date refer to components of costs or resource use modelled as bivariate or multivariate normally or lognormally distributed data (Hahn and Whitehead, 2003; Lambert *et al*., 2008). Lambert *et al*., also illustrate the possibility to increase the flexibility using a two-part model for cost components with excess zeros (Lambert *et al*., 2008). The four-part model in Duan (1983) (see Section 3.1.6) is also in essence a data components model.

Analytical extensions to model components of healthcare resource use simultaneously include: a bivariate Poisson distribution (Cameron and Johansson, 1998), negative binomial marginal distribution and copula functions (Cameron *et al*., 2004), independent Poisson distributions with conditional mean functions that depend on correlated latent effects (Chib and Winkelmann, 2001), a multivariate over-dispersed Poisson mixture model (Gurmu and Elder, 2000), bivariate Poisson-lognormal mixture and bivariate negative binomial regression models (Munkin and Trivedi, 1999), and a bivariate zero-inflated binomial regression for count data with excess zeros (Wang, 2003). These models currently represent possibilities for research, and, although better fit to data is often reported, there is limited evidence on whether they may overfit data and on efficiency of the estimators.

#### 3.1.11. Methods based on averaging across a number of models

Another recent area of research has explored whether averaging results across a number of parametric models (the model averaging approach) could lead to better performance when modelling resource use and cost data given that a priori knowledge of a single appropriate model is not available. Conigliani and Tancredi (2006; 2009) showed that the performance of Bayesian model averaging depends on what models are averaged over and whether there was a model included that fits the data well; otherwise an approach based on mixtures of distributions seemed more appropriate in terms of coverage.

#### 3.1.12. Markov chain methods

An approach based on a finite Markov chain is suggested to estimate resource use over different phases of health care (Coxian phase-type distribution) and evaluate total cost by attaching unit costs to these phases (Marshall *et al*., 2007). A way to implement adjustment for covariates through Bayesian belief networks is also proposed (Marshall and McClean, 2003). An implementation of the approach in evaluating mean length of stay shows better in-sample fit of a phase-type distribution compared with lognormal and gamma models (Faddy *et al*., 2009). This dynamic modelling approach could be very flexible but relies on sufficient data to allow robust modelling and estimation. Also, although the approach was applied to two data sets, more research is needed into its robustness and efficiency in terms of evaluating (conditional) means.

### 3.2. Ability of the identified analytical approaches to address the characteristics of healthcare resource use and cost data

Table I summarises our judgement with respect to the ability of the identified analytical approaches to account for the skewness, heavy tails, excess zeros and multimodality in data, under the adopted analytical categorisation.

**Table I:** Summary characteristics of the reviewed methods for modelling cost and resource use data in moderate size data

	Features of data				Features of method				
		
Analytical approach	Skewness	Heavy tails	Excess zeros	Multimodality	Testing for cost difference	Covariate adjustment	Analysis on original scale/No need to back transform	Works with small samples*	Ease of implementation
I. Methods based on the normal distribution	L	L	L	L	H	H	H	L	H
II. Methods following transformation of data	H	H	L	L	P	H	L	P	P
III. Single-distribution generalized linear models (GLM)	H	P	L	L	H	H	H	P L^a^	H
IV. Parametric models based on skewed distributions outside the GLM family	H	P	L	L	H	H	H	P L^b^	P
V. Models based on mixture of parametric distributions	H	H	P	H	P	H	H	P	L
VI. Two-part and hurdle models	H	P H^c^	H	L	P	H	L H^c^	P L^c^	P H^c^
VII. Survival (or duration) methods									
(i) Semi-parametric Cox and parametric Weibull proportional hazards model	H	H	H	P	P	H	H	P	H
(ii) Aalen additive hazard model	H	H	L	P	P	P	H	L	L
VIII. Non-parametric methods:									
(i) Central Limit Theorem and Bootstrap methods	P	P	L	L	H	H	H	P	H
(ii) Non-parametric modified estimators based on pivotal statistic or Edgeworth expansion	H	P	L	L	P	L	H	P	H
(iii) Non-parametric density approximation	H	H	H	H	P	H	H	L	P L^d^
(iv) Quantile-based smoothing	H	H	H	H	H	H	P	L	L
IX. Methods based on data trimming	L	L	L	L	L	L	H	P L^e^	H
X. Data components models	H	P	P	H	P	H	H	P L^f^	L
XI. Model averaging	H	H	P	H	P	H	H	L	L
XII. Markov chain methods	H	H	P	H	P	P	H	L	L

H, High applicability; P, Possible applicability; L, Low applicability.

* Small sample refers to tens to a few hundreds of participants.

a More complex GLMs require large sample sizes, as does checking parametric modelling assumptions.

b Checking parametric modelling assumptions needs large sample size.

c The ability to model heavy tails depends on the model used in the second part. If the model used in the second part is from Category II ‘Methods based on normality following a transformation of the data’ back transformation will be needed. Checking modelling assumptions needs reasonable sample size. The ease of implementation depends on the models used in the two parts.

d These approaches are not available in standard statistical software.

e Depends on data; in small samples opportunities to check parametric model assumptions are restricted.

f Models beyond those relying on multivariate normality will need large data sets.

More detail is provided in the review templates in the web appendix at http://www.herc.ox.ac.uk/downloads/support_pub.

#### 3.2.1. Skewness and heavy tails

The use of initial transformation of the data or explicit use of skew parametrics distributions (GLM family and other distributions) can allow appropriately for skewness in data. Right tails, heavier than those of the normal distribution, are often observed with resource use and cost data. Manning and Mullahy (2001) report more efficient estimates of OLS models on log-transformed data (compared to a GLM with log link) in the case of large log-scale variance or heavy tail log-scale residuals. On the other hand misspecification of the shape of the distribution can reduce the efficiency of the OLS model on log-transformed data. Extension of the GLM to a Generalised Gamma model (that incorporates lognormal model as a special case) performs well on heavy-tailed data even when the log-error is asymmetric (Manning *et al*., 2005). If the link function is not known *a priori* the Extended Estimating Equations model might be suitable alternative but it needs large samples (Basu *et al*., 2006; Hill and Miller, 2010).

The approaches based on mixtures of distributions, data components or model averaging seem to add further flexibility in acknowledging difficulties in explicitly modelling the skewness in the data. The Cox proportional hazards semi-parametric model is shown to perform well, at least when the proportional hazards assumption is met, presumably due to the non-parametric evaluation of the underlying baseline hazard. The Markov chain methods are also suggested to provide flexibility in modelling skewed, heavy-tailed longitudinal data.

#### 3.2.2. Excess zeros and multimodality

Two-part models seem to outperform other methods when excess zeros are present in data, although models based on mixtures of distributions (Deb and Trivedi, 2002), non-parametric density approximation and proportional hazards methods are shown to perform well in some data sets. It is generally unclear what number or proportion of zeros in data would deem the use of these approaches desirable. When more general multimodality is present, methods based on mixtures of distributions, modelling the whole distribution of the data through a non-parametric density approximation, and modelling different data components or Markov chain methods seem promising approaches.

### 3.3. Other characteristics of the analytical approaches

#### 3.3.1. Adjustment for covariates and need to back transform to the original scale

In randomised trials, it is important to be able to adjust for covariates to gain precision in estimating the mean cost. Most of the identified approaches allow adjustments for covariates to be incorporated. The analytical approach based on initial transformation of the data has an added issue that transformation back to the original scale is needed.

#### 3.3.2. Sample size implications

Most of the analytical approaches identified have been applied to samples of a few hundreds or thousands of observations, and their performance in more limited samples is unclear. Some of the more complex approaches are clearly tailored for the situations when sufficient data are available to inform them. The extended GLM approach proposed by Basu (2005), for example, is not recommended for samples smaller that 5000, which would preclude its use in the majority of randomised trials. Further research is needed to study the performance of approaches based on mixtures of distributions, model averaging and data components in small samples. What is apparent is that what might be considered a sufficient sample size is an empirical issue that will depend on the extent to which skewness, heavy tails, excess zeroes and multimodality are present in the data. In circumstances where few of these issues exist then a sample size in the hundreds may be large enough, but where these issues are all present then a sample size of thousands may nevertheless be too small.

#### 3.3.3. Ease of implementation

Approaches were judged ‘easy to implement’ if available in standard statistical software. These include standard OLS, methods based on alternative distributions with and without initial data transformations, the two-part models and the proportional hazards approaches. Most of the more flexible analytical methods identified in this review are not readily available and require expertise in statistical modelling and computation. These include approaches based on mixtures of distributions, non-parametric density approximation, data components, model averaging and Markov chain methods.

### 3.4. Comparing the performance of the analytical approaches

Many of the studies reviewed included an informal comparison of different methods and approaches applied to the same data (Austin *et al*., 2003; Briggs and Gray, 1998; Buntin and Zaslavsky, 2004; Chen and Zhou, 2006; Cooper *et al*., 2007; Lipscomb *et al*., 1998; Manning and Mullahy, 2001; O'Hagan and Stevens, 2003; Zhou, 2002). O'Hagan and Stevens (2003) suggests that methods based on asymptotic normality or simple bootstrap procedures could be inefficient in the case of skewed data and recommends approaches that explicitly account for skewness (e.g. a lognormal distribution). Adjusted bootstrap approaches to account for skewness (Barber and Thompson, 2000) and to approximate the pivotal statistic (Tu and Zhou, 2000) are also suggested. The proportional hazards method was used as one of the compared models in a number of papers (Austin *et al*., 2003; Basu *et al*., 2004; Dudley *et al*., 1993; Lipscomb *et al*., 1998). This model shows good performance when the proportional hazards assumption holds; a test for proportional hazards is suggested by Basu and Manning (2006). A number of publications compare different models (Austin *et al*., 2003; Basu *et al*., 2004; Briggs and Gray, 1998; Buntin and Zaslavsky, 2004) without recommending a particular approach.

However, twenty of the papers identified included a more formal evaluation and comparison of approaches, in the controlled environment of simulated data. A recent study suggests that, contrary to earlier work, the Central Limit Theorem-based methods and the bootstrap perform well in small data sets with substantial skewness (Nixon *et al*., 2009). Other studies have shown that methods based on initial transformation perform well only when this transformation was appropriate for the data (Briggs *et al*., 2005) and that extended GLM methods are likely to outperform the standard GLM and approaches based on initial data transformation (Basu 2005; Basu *et al*., 2006; Manning *et al*., 2005). A few studies have shown that approaches based on mixtures of parametric distributions outperform single distribution alternatives in the case of heavy-tailed data (Conigliani and Tancredi, 2009; Deb and Burgess, 2003; Deb and Trivedi, 1997).

## 4. DISCUSSION

### 4.1. Scope of the review

We aimed to review the methods currently available to evaluate mean healthcare resource use and costs, likely to be relevant to randomised studies. Clearly, the process of identification of relevant studies to include is not straightforward. While many studies report some analysis of resource use and cost data, these analyses rarely aim to contribute to developing analytical methods. We have employed a combination of literature searches and key researcher contacts to try and ensure that we have not missed important contributions to the analytical approaches.

We have excluded some statistical methods judged beyond the scope of the review. These include panel data methods that are often employed in econometrics to control for unobservable (longitudinal) individual effects constant over time, instrumental variable approaches that are used to model the selection bias when evaluating treatment effects based on non-experimental data, selectivity models as these aim to adjust for selection biases that are usually resolved by the randomisation process in trials, and parametric, semi-parametric and non-parametric methods not focusing on estimation of the mean (such as ordered probit/logit, grouped data regression, multinomial logit, nested logit/probit, kernel-based estimators, quantile regression not aimed at mean estimation) (Jones, 2000).

### 4.2. Extensions to cost differences and cost-effectiveness in trials

Two main approaches towards comparing means are often employed. First, direct comparison of means and their uncertainty when no adjustment for covariates is present is widely used. Second, allocation to treatment as a covariate in a regression model is considered. In this case, direct estimation of mean cost difference is obtained only in the case of a linear model where treatment allocation is represented as an additive effect on the original scale of data; otherwise transformations to the original scale are needed. A further application of the covariate adjusted cost models reviewed here is as prediction equations for the input parameters to decision analytic models. In such situations predictive validity will be very important.

Extension to cost-effectiveness analysis implies the ability to set the analysis of costs and health benefits in a correlated bivariate framework. Few of the reviewed papers explicitly considered extensions to cost-effectiveness. Approaches from other published work suggest that this is done either by considering net benefit, by non-parametric bootstrapping, or by setting up an explicit statistical model that links costs and effects. As the net-benefit could have very different distributional properties over ranges of willingness to pay values, this creates considerable problems for efficient analysis. The bootstrap approach involves estimating the mean incremental cost (by whatever method is chosen) and the mean incremental effect for each bootstrap sample; the succession of estimates provides a distribution of points on the cost-effectiveness plane from which cost-effectiveness measures such as the incremental cost-effectiveness ratios and cost-effectiveness acceptability curves can be estimated. Setting up an explicit statistical model that links costs and effects has proved challenging beyond the bivariate normal situation (in which the incremental costs and effects follow a bivariate normal distribution). Any extensions of this approach so far (i.e. two-part or mixtures approaches) have been implemented using Markov Chain Monte Carlo (MCMC) methods, since maximum likelihood solutions may be difficult to obtain (Lambert *et al*., 2008).

## 5. GUIDANCE TO ANALYSTS BASED ON THIS REVIEW

Most of the methods identified in the review have undergone limited testing in different situations and their use in practice is very restricted. Therefore, no detailed guidance can be provided. In making practical recommendations, we have not advocated many methods suggested in the literature. We have dismissed the truncation methods of Marrazzi since their main aim is to be robust to contaminant outliers, while we regard these ‘outliers’ as being part of the true distribution of costs. Other models that have been used in the papers we reviewed appear too complicated for randomised trial data of usually fairly small sample size, have only been shown to be of (sometimes rather slight) benefit in particular data sets, and present formidable problems of implementation to applied analysts. We have outlined three groups of methods, which we term ‘orbits’.

### 5.1. The green orbit

In the green orbit, we can apply simple methods because we have enough data. Analysis can be based on assuming normal distributions for costs. Their underlying true distribution will of course not be normal, but the analysis will depend only on sample means and variances. How do we know when we are in the green orbit? The sample size must be large enough for a number of possible problems to disappear.

- Despite skewness, excess zeros, multimodality and/or heavy tails, the samples should be big enough for the Central Limit Theorem to guarantee near-normality of sample means. How large this is depends on the degree of skewness and also on the complexity of the covariate adjustment or subgroup analysis that is to be performed.
- The number of large costs should be sufficient for the answers not to be unduly influenced by a few very large outlying costs. This is related to the last point, but merits separate checking.

Whether we use frequentist or Bayesian analyses does not matter (because any prior information should be weak enough to be overwhelmed by the data), and so is a matter of personal preference. In broad terms, to be sure we are in the green orbit, we should certainly have several hundreds of observations in each treatment group (or sub-group), and possibly thousands.

### 5.2. The amber orbit

In the amber orbit, we can still use relatively simple methods, but checking the sensitivity of the results to assumptions is recommended. These methods will generally be able to deal with one or two of the possible complications with cost data, but any increase in complexity rapidly takes us into the red orbit.

#### 5.2.1. Alternative distributions

Where the data are skewed and/or heavy tailed, we can model the costs using appropriate alternative distributions instead of assuming normality. Gamma distributions are not recommended because they are sufficiently light tailed that the answers will often be similar to using normal distributions (Manning and Mullahy, 2001). Inverse gamma or lognormal distributions may often be appropriate, but particularly in the case of the lognormal distribution the results may be non-robust to outliers in the data. The log-logistic distribution may be too heavy tailed to often be realistic in practice. Where there are enough data, or background knowledge, to suggest a particular form of distribution, then analysis using this distribution can be recommended, but sensitivity to alternative choices of distribution should be assessed (Nixon and Thompson, 2004).

#### 5.2.2. Transformations

We can also consider transforming the costs to reduce skewness, so that normality can be assumed on the transformed scale. Using the log transform comes with the same caveat about non-robustness to outliers as lognormal models. (It also cannot be used if there are zero costs in the data; the device of replacing zero by a small number is not recommended.) Other power transformations may be considered. However, it is essential that an appropriate ‘back transformation’ is used to produce inferences on the original cost scale, rather than on the transformed scale. Checking sensitivity to the choice of transformation is recommended.

#### 5.2.3. Generalised linear models

GLMs are an attractive approach when we have covariates, because they offer some of the benefits of alternative distributions and/or transformation without the need to back transform. Limitations of GLMs are that they are based implicitly on assuming a particular distributional form (and so there is again a recommendation to check for sensitivity to this choice), and that the frequentist inferences involve approximation. Also, unless the identity link function is used (which may not always be realistic) there is still a back transformation issue that can lead to substantial loss of precision from ignoring the fuller characteristics of the data generating process.

#### 5.2.4. Two-part or hurdle models

These models specifically address zero costs, since the presence of zero values in the data is usually incompatible with any assumed continuous distribution of costs. When modelling resource use with discrete distributions, zeros may be expected, but two-part modelling may still be indicated to deal with large numbers of zeros.

### 5.3. The red orbit

Methods in the red orbit are more complex and generally require substantial expertise, both in statistical modelling and in computation. Many of these approaches are relatively (or even completely) untried in practice. For highly complex models, the only practical computational tool to obtain results is Bayesian MCMC methods. The substantial experience needed to use software for Bayesian analysis and MCMC means that these methods are inevitably in the red orbit. We indicate here some other typical combinations of circumstances that characterise being in the red orbit.

- When addressing skewness through alternative distributions or transformations, the presence of covariates or censoring will typically mean that suitable analyses are complex and not available in standard software.
- Multimodality may suggest either decomposing costs into resource use components, or else using mixture models so as to link the prevalence of different mixture components to covariates (in the same way as is usually done for the prevalence of zeros in two-part models). Another use for mixture models is to allow the tail thickness of the cost distribution to be fitted separately from the main body of data. Both resource use components models and mixture models are sufficiently complex to belong in the red orbit.
- Where the analysis involves comparing different treatment groups or sub-groups, there will typically be information to suggest some similarity between parameters of the different groups. This kind of structural prior information may be modelled naturally in a Bayesian framework, and can be influential in the analysis even when more quantitative prior information is lacking. The resulting Bayesian hierarchical models are in the red orbit.

Any suggested practical strategy comes with its own health warnings. It is based on our interpretation and opinion of the current literature, and others may disagree. The recommendations may be modified, or even overturned, by future research. Moreover, for any particular data set, it is likely that special methods may be found which out-perform those suggested here; our intention is only to give a strategy which we believe should have wide applicability. This initial guidance will evolve over time with future research likely to enable more detailed guidance.

## 6. FUTURE RESEARCH

Further research comparing the performance of different methods on simulated as well as experimental trial data is highly desirable. The literature review has suggested that mixture models could have significant advantages in modelling skewed, heavy-tailed, multimodal data. Although a number of applications have exemplified models of healthcare resource use employing this method, further applications are needed to support its wider use. There are methods for analysing cost data that we believe hold some promise for the future, but cannot at present be recommended for applied work. One is model averaging approaches which make some allowance for the uncertainty in choosing an appropriate statistical model (Conigliani and Tancredi, 2009). A second is models which allow for potentially heavy upper tails, while allowing flexible (or even non-parametric) distributions for the bulk of the data (Conigliani and Tancredi, 2005). A third is Bayesian approaches which incorporate informative priors about distributional shape, particularly about the upper tails (O'Hagan and Stevens, 2003). The latter is in contrast to models, implemented in MCMC simply for computational convenience, and that in general use priors intended to be non-informative. So far, data component models have not been shown to lead to any improvement in efficiency and face significant technical difficulties but the research in this area is limited (Hahn and Whitehead, 2003; Lambert *et al*., 2008).

A major limitation of the implementation of more complicated models in the field of randomised trials is the need for the analytical framework to accommodate both costs and health effects and evaluate the summary cost-effectiveness measures. In doing so, the analysis should allow for the correlation structure of different outcomes. The future development of such approaches in different situations is recommended, perhaps especially for two-part models or mixture models.
